# Best Available Evidence in Cochrane Reviews on Herbal Medicine?

**DOI:** 10.1155/2013/163412

**Published:** 2013-06-12

**Authors:** Elyad Davidson, Julia Vlachojannis, Melainie Cameron, Sigrun Chrubasik

**Affiliations:** ^1^Pain Relief Unit, Department of Anesthesia, Hadassah Hebrew University Hospital, Jerusalem, Israel; ^2^Institut für Rechtsmedizin, Universität Freiburg, Albertstrasse 9, 79104 Freiburg, Germany; ^3^School of Health and Sport Sciences, Cluster for Health Improvement, University of the Sunshine Coast, Maroochydore DC, QLD 4558, Australia

## Abstract

Cochrane reviews are considered by many to be the “gold standard” or the final word in medical conversation on a topic. We explored the eleven most relevant Cochrane reviews on herbal medicine and identified that frequently herbal medicines in the included studies had not been sufficiently well characterised. If data on the effects of the plant parts are unavailable, effects of co-active ingredients need to be considered and the plausibility of the study medications for the specific indications discussed. Effect sizes calculated from exploratory studies would be best used to determine the sample sizes required for future confirmatory studies, rather than as definitive reports of intervention effects. Reviews should be comprehensive, including discussion of putative adverse events and possible drug interactions. We suggest that the guidelines for preparing Cochrane reviews be revised and offer assistance in this task.

## 1. Introduction

The Cochrane Collaboration was established in 1993 as an international network that now includes more than 28,000 people from more than 100 countries. The aim of the Cochrane Collaboration is to help health care providers, policy-makers, and patients and their advocates and carers, to make well-informed decisions about health care based on the best available research evidence. Collaboration members prepare, update, and promote “Cochrane reviews”—presently about 5000 reviews are published in “The Cochrane Library” (http://www.cochrane.org/about-us). The combination of high quality work, conservative approaches to clinical decision-making, and open access publication has led to Cochrane being regarded as the final word in the medical debate on a topic (i.e., gold standard, http://www.hsl.virginia.edu/collections/ebm/aboutcochrane.cfm). 

This standard may hold true for Cochrane reviews on medical interventions including synthetic medications. Herbal medicines, however, differ from chemical entities in so far as they contain many ingredients (sometimes huge numbers) that may have additive, synergistic, or partially or fully antagonistic activities if they are co-active [1]. Resultant effect is caused by “the active principle” of a herbal medicine, that is, the combination of all active ingredients. If herbal medicines are mixed, interactions may increase, making it more difficult to understand the complexity of the resultant effect. Authorities including the World Health Organization and the European Medicines Agency (EMA) have published guidelines for assessing the quality of herbal medicines (http://apps.who.int/medicinedocs/documents/s14878e/s14878e.pdf, http://www.ema.europa.eu/docs/en_GB/document_library/Scientific_guideline/2009/09/WC500003370.pdf). 

The aim of this study was to explore new or updated Cochrane reviews on herbal medicines for quality of reporting as an exercise to evaluate whether these publications represent the best evidence. CONSORT guidelines for clinical trials are the reporting standards recommended by the Cochrane Collaboration [2]. The recommended quality items to be considered when reporting trials on herbal medicines are summarized in the elaborated CONSORT statement that has been published in 2006 [2]. 

## 2. Methods

The Cochrane Database of Systematic Reviews “Issue 1 of 12, January 2013” was searched using the term “herbal medicine” as “Title, Abstract, or Keywords,” sorted by relevance (not alphabetical or date). Two authors (Sigrun Chrubasik and Julia Vlachojannis) extracted the data independently, recording the number of studies with confirmatory study design or intention-to-treat (ITT) analysis as a proportion of included studies. Confirmatory studies were defined as those that tested hypotheses and include *a priori* power and sample size calculations (a power, or alpha level, of 80% (90%) being associated with a 20% (10%) risk of Type II error, or failure rate to detect a difference, beta level). ITT analysis includes data from all possible participants, even those who did not complete the intervention. Missing data are replaced either by inserting baseline values or carrying forward the last values, in order not to favour the intervention over the control. Other details extracted were the duration of the studies, outcome measures considered, number and types of herbal medicines studied, and the herbal medicine characteristics (plant parts stated, dose per day, in case of extract the drug extract ratio and solvent, and content of marker substances in the daily doses). Disagreements in data extraction were resolved by discussion and consensus (all authors). 

## 3. Results

There were 102 reviews on “herbal medicine” identified from 7694 records of title, abstract, and keywords in the Cochrane library. Reviews were sorted by relevance, and we selected the top eleven reviews for analysis (we had planned to include the 10 most relevant reviews, but one of these had zero articles included, thus we extended our examination to include the next most relevant review). Most of these reviews included studies investigating Chinese herbal medicines (see Table 1). The plant parts used to prepare the study medications and the daily doses of the herbal medicines were rarely stated; likewise in case of extracts, drug: extract ratios, and solvents, and for only few of the herbal medicines were the quantities of marker substance(s) per day given.  Table 2 summarizes the trial quality details of the data extraction. The reviews included zero (no studies; see [10]) to 75 clinical studies (see [3]). Most studies were of exploratory study design (*n* = 251). Only nine studies had confirmatory study designs. Not all outcome measures selected in the herbal medicine trials and reviews appeared to be meaningful or logical. For example, duration of the included studies did not exceed 6 months (mean duration across all studies was 2.6 months) in one of the reviews that reported mortality as primary outcome measure [6]. Similarly, despite short mean observation periods of 54 days or 2 to 3 months, mortality or cardiovascular events were chosen as primary outcome measure in two other reviews [4, 13]. Plausibility for the plant parts used for specific indications was not reported or discussed in any review; neither were putative interactions and adverse events given consideration.

## 4. Discussion

### 4.1. Characterisation of the Study Medication

The World Health Organisation (WHO) recommends that the manufacturing procedure of medicinal plant parts should be described in detail (http://apps.who.int/medicinedocs/en/d/Jh2984e/). Identification of the plant part and, where possible, assay of the plant preparation should be reported. If identification of an active principle is not possible, it may be sufficient to identify a characteristic substance or mixture of substances (e.g., “chromatographic fingerprint”) to ensure consistent quality of the preparation, such that the study may be repeated with an “essentially similar” product [14]. Without this information, study results can be attributed only to the plant part used in the individual study and cannot be generalized because starting materials may vary considerably in the composition of the active principle. Declaration of marker substances for every plant part included in a product may help manufacturers to produce somewhat similar products; however, marker substances were stated in only 8 of the 326 included herbal medicines in this exercise. Another possibility is to prepare the herbal products according to the Guideline of Good Manufacturing Practice (http://ec.europa.eu/health/documents/eudralex/vol-4/index_en.htm). In the review on herbal medicines for the treatment of irritable bowel syndrome (IBS) [3] only two of the 75 herbal medicines were prepared according to the WHO recommendation and commercially available: Iberogast^R^ [15] and Padma Lax^R^ (http://www.swissmedic.ch/bewilligungen/00009/00011/index.html?lang=de, see Betriebsbewillungen). 

We cannot determine whether comparable quality standards are considered in patented Chinese herbal products (see Table 1). For most Chinese herbal medicines, neither the Latin binomial name of the plant and the part of the plant, nor the dose used, were reported. For example, the composition of the mixture “Daming,” used for lowering elevated serum cholesterol [13], is described as consisting of *Rheum palmatum*, *Cassia obtusifolia*, *Salvia miltiorrhiza*, and *Panax ginseng* in the ratio of 12 : 12 : 6 : 1  [16]. These plant parts were not stated in the publication of the clinical trial, nor were they included or discussed in the review. Based on common usage, we might assume that the roots of rhubarb and ginseng, the fruit or leaf of *Senna *(*Cassia*), and the herb (whole, above-ground plant) of sage were used. Three of these plant parts contain toxic compounds (hydroxyanthracene glycosides, monoterpenes; see section on adverse events), but the lack of details in the study included in the Cochrane review means that we cannot be sure about the quantity of toxic components in the tested study medication Daming. Likewise, the study medication “Xiaozhiling” has been described as “a mixture of herbs” [13]. The only Xiaozhiling formulation we found detailed in the public domain was for injection into tumours (http://www.gp-tcm.org/wp-content/uploads/2009/11/D5.4-Vol-III-FINAL.pdf?rs_file_key=9133909644e22b12f07b2e994153490). We cannot be certain whether the product used for the treatment of hypercholesterolaemia [13] was essentially similar. In some studies, herbalism is practiced in a dynamic fashion—that is, up to 23 different herbal medicines have been administered concomitantly (see Table 1) and the formulae changed in the course of the study [11]. The results achieved with original and changed formulations cannot be considered together because they provide different active principles.

According to the CONSORT checklist, reporting such information, including the Latin names, plant parts, doses, and marker substances, is mandatory. Correct identification of plants and use of nomenclature are also required. For example, in reviewing herbal medicines for low back pain, Gagnier et al. [5] attributed the results of studies that investigated extract from willow bark to *Salix alba* (White willow); however, the proprietary extract investigated in these studies was obtained from *Salix purpurea *(Purple willow) [17, 18]. According to the WHO guideline (http://apps.who.int/medicinedocs/en/d/Jh2984e/), herbal medicines are defined as being finished, labeled medicinal products that contain as active ingredients aerial or underground parts of plants, or other plant material, or combinations thereof, whether in the crude state or as plant preparations. Plant materials combined with chemically defined active substances (synthetic or isolated constituents of plants) are not considered to be herbal medicines; however, this stringent definition of herbal medicines appears only to have been applied to very recent Cochrane reviews on herbal medicines [19, 20]. For example, a study investigating an ointment containing isolated or synthetic compounds—ethy salicylate, methyl salicylate, glycosalicylate, salicylic acid, camphor, menthol in addition to capsicum oleoresin—should not have been included in the review of herbal medicine for low back pain [5]. Likewise, single ingredients (e.g., the alkaloid berberine [6] or policosanol [13] or the mixture of ispaghula husk and a lipase inhibitor [21]) are not defined as herbal medicines and should be excluded from reviews on herbal medicines for type 2 diabetes, hypercholesterolaemia, and IBS. Further, some Chinese herbal medicines may not solely derive from plants; they may also contain animal products or minerals [7]. We recommend that the Cochrane Collaboration stipulate that studies of such mixed preparations be handled independently, respecting the WHO definition of herbal medicines.

### 4.2. Plausibility of Study Medications

For many medicinal plant parts, the qualitative and quantitative content of ingredients is known, as well as the effects these ingredients produce. An analysis of the plausibility for using particular medicinal plant parts for particular indications is notably absent from all eleven Cochrane reviews considered in this project. For example, one of the trials in the review on IBS investigated a decoction of *Senna* leaf, which was administered together with fluoxetine daily, and clonazepam 1 tablet before bed, over 15 days [22]. Folium sennae is included in the official monographs of the EMA. This medicinal plant part is recommended for short-term use in cases of occasional constipation, and only if a therapeutic effect cannot be achieved by a change of diet or the administration of bulk forming agents (http://www.ema.europa.eu/docs/en_GB/document_library/Herbal_-_Community_herbal_monograph/2009/12/WC500018215.pdf). The EMA monograph recommends that the daily dose of folium sennae should contain no more than 30 mg of hydroxyanthracene glycosides (calculated as sennoside B). This dose is usually contained in 2 g  dried leaf (http://www.mdidea.com/products/new/new04009.html); participants in that study received three times the “occasional constipation” dose in order to reduce IBS complaints [22]. Such high doses are associated with intolerable and inacceptable adverse events (see section adverse events). We maintain that this study should not have been included in the review on IBS due to a probably inappropriate use of a herbal medicine, and poor reporting of potential interactions, side effects, and adverse events.

An additional example is the Chinese herbal medicine “Xuezhikang” [13]. Eighteen studies investigated the effect of “Xuezhikang” on elevated serum cholesterol but no information has been provided explaining the rationale behind this medication. An internet search of public domain resources allowed us to identify that “Xuezhikang” is the ethanolic extract of red yeast rice, a processed product of yeast (*Monascus purpureus*) grown on rice (http://www.naturalstandard.com/index-abstract.asp?create-abstract=redyeast.asp&title=Red%20yeast%20rice). The product contains several compounds collectively known as monacolins, substances that inhibit cholesterol synthesis. One of these, monacolin K, has the same chemical structure as the drugs lovastatin and mevinolin, potent inhibitors of HMG-CoA reductase (http://www.naturalstandard.com/index-abstract.asp?create-abstract=redyeast.asp&title=Red%20yeast%20rice). We argue that this information should have been included in the Cochrane review as part of the rationale for the intervention. 

In short, in cases where the active principle of the herbal medicine is not known, we suggest that Cochrane reviews should incorporate a table with co-active ingredients and their known effects and/or discuss the plausibility of the study medication.

### 4.3. Completeness of the Reviews

Some of the reviews might be considered incomplete. For example, the Cochrane review on IBS included preparations from single plant parts only. Reasons for not including the clinical trials using extracted volatile oil of *Mentha piperitum* [23] or with other medicinal plant products (e.g., extracts from *Hypericum perforatum* or *Curcuma longa* [24] or psyllium (ispaghula seed or husk)) were not stated. 

Ispaghula seed is the dried ripe seed of *Plantago ovata*. The ratio of soluble (mucilage mucopolysaccharides) to insoluble fibres is 47 : 53. The ESCOP monograph recommends for the treatment of constipation or irritable bowel syndrome in adults and children older than 12 years a daily dose of 7 to 30 g [25]. The episperm and collapsed layers from the seeds of *Plantago ovata*, the ispaghula seed husk, may be used instead in a dose of 4 to 20 g per day [26]. In a dose-finding study [27], 20 g  ispaghula seed husk was identified as possibly optimum daily dosage for treating IBS complaints. Lower doses were less effective. Only one of the clinical trials [28] included in the Cochrane review on bulking agents for IBS [29] studied a daily dose of 20 g  of the husk. Ruepert and colleagues' general conclusion that there is no evidence that bulking agents are effective for treating IBS is not justified unless the optimum dose for soluble fibres for the relief of IBS complaints has been predetermined. Unfortunately, the most recent trial investigating psyllium seed was also underdosed at 10 g  of psyllium per day [30]. 

Further, another Cochrane review on the effectiveness of artichoke leaf extract for treating hypercholesterolaemia [31] had identified 3 clinical trials. Only one of these studies has been considered in the review by Liu et al. [13]. Rationale for excluding the study by Englisch et al. [32] was given as “lack of randomisation,” but identifying this study as nonrandomised might not be justified because this study was conducted in Germany where the International Conference on Harmonisation Good Clinical Practice (ICH GCP) guidelines were recommended from 1986 onwards and constituted in 1989. It could be assumed that a study carried out in 1996 with ethics committee approval was compliant with the ICH guidelines regarding randomisation, allocation concealment, and blinding of participants and assessors. For this reason this same study was included in the Cochrane review by Wider et al. [31]. (Wider, and at least 2 of her colleagues, are German, and completely au fait with the German law.) The evidence of effectiveness of artichoke leaf extract for hypercholesterolaemia was, thus, underreported in the review by Liu et al. [13]. 

### 4.4. Exploratory Studies and Effect Sizes

Typically, effect sizes are calculated for all studies included in Cochrane reviews. Hypotheses and sample size calculations are proposed items of the CONSORT checklist [2]. Only studies with a confirmatory study design that consider possible confounders in their analysis demonstrate efficacy. Exploratory studies are likely to be underpowered and at increased risk of Type II error; thus we recommend that effect sizes are reported so that likely effects will not be overlooked. Effect sizes from exploratory studies present numbers that cannot be relied upon to predict clinical outcomes, but they are helpful in calculating sample sizes for future confirmatory studies. Only 3 of the 209 clinical trials had hypotheses, and none of these were stated in the articles. It remains unclear if the hypotheses were clinically relevant. 

### 4.5. Putative Adverse Events and Interactions

Although major adverse events did not occur in any of the short-term clinical trials, putative adverse events could be discussed in light of the fact that the herbal medicines may be taken over longer times than investigated. The study medication “Daming,” for example, will have a laxative effect. Daming could be standardized on the total anthraquinone content, or its main marker compound chrysophanol [16], but the total anthraquinone content in the daily “Daming” dosage was not stated. Because anthraquinone derivatives are toxic compounds [33, 34], and sage contains toxic monoterpenes [35], we maintain that a warning should have been included in the review not to use “Daming” for long-term treatment. The ESCOP monograph limits use of oral sage (infusions of 3 g) to up to 4 weeks.

Adverse events were not reported in the study investigating *Senna* leaf for IBS [21]. *Senna* leaf may produce abdominal pain and spasm and passage of liquid stools, in particular in patients with irritable colon. It may well be that the patients received clonazepam concomitantly to treat the putative abdominal complaints. Also, details of patients' concomitant medications for other diseases were not reported in this study. Hypokalaemia resulting from long-term laxative abuse (i.e., more than 15 days continuous use of the EMA recommended dose of *Senna* leaf) may potentiate the action of cardiac glycosides and interact with antiarrhythmic medicinal products (prescribed to induce reversion to sinus rhythm, e.g., quinidine, or prolong QT-phase). Concomitant use of *Senna* leaf preparations and other medicinal products inducing hypokalaemia (e.g., diuretics, adrenocorticosteroids, and liquorice root) may disturb electrolyte balance. 

 Further, the Cochrane review on St. John's wort for depression [36] did not raise the importance of the hyperforin content in medicinal plant products. Hyperforine is the *Hypericum* ingredient which is related to St. John's wort drug interactions [37].

## 5. Conclusion

Cochrane reviews on herbal medicines should make transparent exactly what was studied and allow readers to accurately compare studies. We suggest that the reviews should report a table of detailed study medication characteristics including the Latin names of the plants, the plant parts, the preparation investigated (crude drug, extract, and if extract drug extract ratio and solvent), and daily dose. If known, then further details, such as the proprietary product or extract name, the manufacturer, and the daily dose of marker substance(s), should be added to improve the repeatability of studies. Formulae changes of herbal mixtures differ in their active principles from the original formulations, and should be handled as separate medications. Studies with single compounds or mixtures with nonherbal entities should be excluded from reviews on herbal medicines. 

Plausibility for the choice of the plant part(s) and the dose with respect to the medical indication should be checked. Effect sizes calculated from exploratory studies are best used to determine sample sizes required in planning future confirmatory studies. In the interest of clinical safety, we recommend that putative adverse events and possible interactions also be discussed. In at least some Cochrane reviews on herbal medicine the best available research evidence could be improved to maintain the claim that Cochrane reviews are the gold standard for evidence-based medicine. We hope our discussion, as well as proposals and annotations, stimulate re-thinking of the guidelines for the preparation of Cochrane reviews of herbal medicines in order to make them more transparent and useful for health care providers, policy-makers, and patients and their advocates and carers.

## Figures and Tables

**Table 1:** The eleven most relevant Cochrane reviews extracted for the most important details of herbal medication characteristics.

Reference	Origin of herbal	Herbal medicine characteristics				
	medicines	Plant part (PP)	mg/day	DER	Solvent	Marker/day
[3] (5/2011) Irritable bowel syndrome	66 Chinese 1 Tibetan 1 Indian 3 European	Stated in *n* = 3 2 patented products up to 20 PP				

[4] (9/2012) Advanced colon cancer	Chinese cotreatment to chemotherapy	Not stated				

[5] (4/2006) Low back pain	European^$^	Stated in *n* = 8	Stated in *n* = 8	Stated in *n* = 8	Stated in *n* = 8	Stated in *n* = 8

[6] (1/2009) Type 2 diabetes	69 Chinese^&^	Stated in *n* = 2 up to 20 PP				

[7] (10/2005) Schizophrenia	2 Chinese 2 Chinese + Western 1 European + Western	Stated in *n* = 3^#^ up to 10 PP formula changes in *n* = 1	Stated in *n* = 3			

[8] (5/2012) Threatened miscarriage	5 Chinese 20 Chinese + Western	Stated in *n* = 3 up to 14 PP formula changes in *n* = 28				

[9] (1/2010) Chronic neck pain	Chinese	Stated in *n* = 2 up to 6 PP	Stated in *n* = 1			

[10] (4/2006) Pre-eclampsia	zero					

[11] (11/2010) Primary dysmenorrhea	Chinese	Stated in *n* = 37 up to 23 PP formula changes in *n* = 19	Stated in *n* = 10			

[12] (6/2011) Diabetic neuropathy	Chinese^%^	Not stated	Not stated			

[13] (8/2011) Hypercholesterinaemia	3 Chinese^§^ 1 European	Stated in *n* = 1	Stated in all			

DER: drug extract ratio; ^&^one containing an additional alkaloid; ^#^extract Egb761 is produced according to GMP from Ginkgo leaf;  ^§^one study investigated an isolated compound; ^$^two external medications containing additional synthetic compounds; ^%^eight patented Chinese products.

**Table 2:** The eleven most relevant Cochrane reviews on herbal medicines extracted for confirmatory study design, ITT analysis, study duration, outcome measures, and numbers of different study medications.

Reference	No. of studies	Confirmatory studies	ITT analysis	Study duration	Outcome measures		No. of study medications
					Primary	Secondary	
[3] (5/2011) Irritable bowel syndrome	*n* = 75	*n* = 3 Not stated if hypothesis clinically relevant	*n* = 3	9 days to 18 wks	Global improvement of symptoms Quality of life	No. of recurrent episodes Predominant symptom Cost-effectiveness Adverse events	*n* = 71

[4] (9/2012) Advanced colon cancer	*n* = 20	*n* = 0	*n* = 0	7–126 days Mean 54 days	Mortality**	Survival time Relative response	*n* = 16 Oral *n* = 11 Injection *n* = 5

[5] (4/2006) Low back pain	*n* = 10	*n* = 4	*n* = 7	Mean 3.5 weeks	Pain intensity, function Overall improvement Return to work		*n* = 6 Oral *n* = 3 External *n* = 3

[6] (1/2009) Type 2 diabetes	*n* = 66	*n* = 1 Not stated if hypothesis clinically relevant	*n* = 1	2–6 months Mean 2.6 months	Mortality* Diabetic complications* Quality of life*	Fasting glucose, HbA1C BMI Fasting insulin AEs and costs	*n* = 69 Oral *n* = 68 Injection *n* = 1

[7] (10/2005) Schizophrenia	*n* = 7	*n* = 0	*n* = 0	20 days–6 months	Clinical response subscores	Various	*n* = 5

[8] (5/2012) Threatened Miscarriage	*n* = 44	*n* = 0	No dropouts reported in any study	Not stated in all	Continuation of pregnancy after 28 wks of gestation	During treatment After treatment Foetus	*n* = 25

[9] (1/2010) Chronic neck pain	*n* = 4	*n* = 0	*n* = 2	4 wks	Pain (VAS) Functional status Patient satisfaction	Neurologic outcomes Adverse events	*n* = 3

[10] (4/2006) Pre-eclampsia	*n* = 0	—	—	—	Maternal: for example, death Neonatal: for example, death	Various	—

[11] (11/2010) Primary dysmenorrhea	*n* = 39	*n* = 0	*n* = 0	Up to 6 cycles Approx. 6 months	Menstrual pain Menstruation-related symptoms, AEs	Laboratory values Additional medications Satisfaction	*n* = 39 Oral *n* = 34 External *n* = 3 Rectal *n* = 1 Sublingual *n* = 1

[12] (6/2011) Diabetic neuropathy	*n* = 39	*n* = 0	*n* = 0	At least 4 wks	30% improvement in global score (e.g., VAS)	Quality of life Nerve conduction AEs	*n* = 30 Oral *n* = 26 Injection *n* = 3 Either or *n* = 1

[13] (8/2011) Hyper cholesterolaemia	*n* = 22	*n* = 1	*n* = 1	Mean 2.3 months	Cardiovascular event Serum cholesterol	Death, quality of life Serum triglycerides, BMI, AEs, and so forth	*n* = 5

*In case of 5-year studies; **disease-related death/total recruited patients × 100%.
